# Low-Temperature Flexible Micro Hydrogen Sensor Embedded in a Proton Battery for Real-Time Microscopic Diagnosis

**DOI:** 10.3390/mi12101215

**Published:** 2021-10-05

**Authors:** Chi-Yuan Lee, Chia-Hung Chen, Chin-Yuan Yang, John-Shong Cheong, Yun-Hsiu Chien, Yi-Chuan Lin

**Affiliations:** 1 Department of Mechanical Engineering, Yuan Ze Fuel Cell Center, Yuan Ze University, Taoyuan 32003, Taiwan; s1105010@mail.saturn.yzu.edu.tw (C.-Y.Y.); johnshong1018@gmail.com (J.-S.C.); s1040923@g.yzu.edu.tw (Y.-H.C.); chuan881018@gmail.com (Y.-C.L.); 2 HOMYTECH Global CO., LTD., Taoyuan 33464, Taiwan; chenjahon@gmail.com

**Keywords:** proton battery, micro-electro-mechanical systems, low-temperature micro hydrogen sensor

## Abstract

The proton battery is a very novel emerging research area with practicability. The proton battery has charging and discharging functions. It not only electrolyzes water: the electrolyzed protons can be stored but also released, which are combined with oxygen to generate electricity, and the hydrogen is not required; the hydrogen ions will be released from the battery. According to the latest document, the multiple important physical parameters (e.g., hydrogen, voltage, current, temperature, humidity, and flow) inside the proton battery are unlikely to be obtained accurately and the multiple important physical parameters mutually influence the data; they have critical effects on the performance, life, and health status of the proton battery. At present, the proton battery is measured only from the outside to indirectly diagnose the health status of battery; the actual situation inside the proton battery cannot be obtained instantly and accurately. This study uses micro-electro-mechanical systems (MEMS) technology to develop a low-temperature micro hydrogen sensor, which is used for monitoring the internal condition of the proton battery and judging whether or not there is hydrogen leakage, so as to enhance the safety.

## 1. Introduction

The fuel cell technology has been universally used in extensive domains, but there are still many problems to be solved and overcome, such as the volume and weight of the fuel cell and the storage of hydrogen. Heidari et al. [1] first developed a proton battery consisting of a hydrogen storage tank + fuel cell. The fuel cell itself has the hydrogen storage function; it is unnecessary to prepare an additional hydrogen storage tank: the risk of hydrogen leakage can be reduced and the space and cost of the hydrogen storage tank can be reduced. In comparison to the proton exchange membrane fuel cell (PEMFC), the proton battery is rechargeable and free from hydrogen and the hydrogen ions will be released from the battery. In the proton battery charging process, the water is decomposed with the assistance of a power supply to generate hydrogen ions, which are stored in porous carbon-based material as a hydrogen storage electrode. Folonari and Condon et al. [2,3] indicated that the new kind of fuel cell composed of hydride electrode and solid electrolyte not only has higher energy density and more compact simple structure but also keeps the advantages of traditional fuel cell. This kind of structure can significantly enhance the reliability and performance of electric vehicles. At present, most mainstream fuel cells store the hydrogen in the hydrogen storage tank, and then the hydrogen storage tank is installed on the vehicle, e.g., automobile and generator. The metal hydride-nitride composed the composite electrode of homogeneous regenerative fuel cell; the storage capacity of hydrogen in the electrode measured in charging mode was 0.6 wt%; the discharged hydrogen volume could be detected, but it was very low, only about 0.01 wt%, inducing safety risk [4]. Jurewicz et al. [5,6,7] used viscose fabric pyrolysis of liquid potassium hydroxide (KOH) electrolyte (concentration 6 M) to prepare activated carbon electrodes for electrochemical hydrogen storage, implemented 1.5 wt% reversible hydrogen storage, and studied the influence of acidity and alkality of electrolyte on hydrogen storage capacity. Babel et al. [8] manufactured a highly porous carbon; the high hydrogen storage capacity of 1.89 wt% proved again that the ultramicropore and micropore volumes and the relatively smaller mesopore volume enhanced the hydrogen storage capacity. Bosch et al. [9] indicated that as the hydrogen storage characteristic of nanostructure of carbon was known, the larger surface area and improved material could enhance the feasibility of rechargeable proton battery with high energy density and high hydrogen storage capacity. The proton battery is a very novel research area: there are some immature prototypes [10,11,12] worth researching. The proton battery has two modes. In the charging mode, water enters the oxygen side and is electrolyzed in the gas diffusion layer (GDL) to produce H^+^ and O_2_. Only H^+^ can be stored on the activated carbon electrode. In the discharge mode, setting a constant discharge current can make H^+^ separate from the activated carbon electrode, and then circulate into the oxygen side and react with oxygen to generate electricity (in the form of water).

A three-dimensional interconnected reticular porous carbon (3D-RPC) with a large surface area of 1535 m^2^·g^−1^ by a sol–gel method followed by carbonation as well as a chemical activation process using corn starch as a carbon precursor was demonstrated. The 3D-RPC shows the ultrahigh specific capacitance of 372 F·g^−1^ at a current density of 0.5 A·g^−1^ in a 2 M KOH electrolyte, and the 3D-RPC//3D-RPC symmetry supercapacitors achieved a high energy density of 9.2 Wh·kg^−1^ as well as long cycle life with the capacitance retention of 90% after 10,000 cycles. Furthermore, [BMIm]BF_4_/AN ionic liquid was selected as the electrolyte to improve the potential operating window where the high specific capacitance of 218 F·g^−1^ at a current density of 0.5 A·g^−1^ can be achieved. A 3D-RPC based symmetry supercapacitor in [BMIm]BF_4_/AN delivers an outstanding energy density of 24.5 Wh·kg^−1^, which is higher than that of most previously reported carbon materials [13,14].

As the proton battery is an emerging research area, the internal physical quantities of proton battery shall be diagnosed instantly to enhance the performance and safety of battery. At present, the data are obtained only from outside of proton battery, so this study uses micro-electro-mechanical systems (MEMS) technology to develop a low-temperature flexible micro hydrogen sensor, which is embedded in the proton battery for real-time microscopic detection, so as to judge whether there is hydrogen leakage to enhance the safety.

## 2. Sensing Principle of Flexible Micro Hydrogen Sensor

The micro hydrogen sensor in this study is a semiconductor sensor. The sensing principle is to use the reducing gas and the oxygen adsorption on the surface of gas-sensing thin film; when the oxygen atom (O) is adsorbed on the surface of gas-sensing thin film, it is likely to capture the electrons in the material to form adsorbates such as oxyanion (O^−^), and the electrons in the gas-sensing thin film are reduced, so that the resistance of sensing material increases. When the surface of the gas-sensing thin film contacts the reducing gases (e.g., CO, H_2_) in the environment, the reducing gas reacts with the oxyanion adsorbed on the surface of gas-sensing thin film, performing oxidation reaction with the oxyanion to release electrons into the gas-sensing thin film, the returned electrons reduce the overall resistance of material.

The maximum adsorbance on the surface of the gas-sensing thin film and the overall resistance of thin film depend on the amount of reducing gas, the operating temperature of gas-sensing thin film, the quantity of adsorption sites and the film surface activity in equilibrium state. The gas sensor has different sensitivity levels for different gases and working temperatures (oxidation rate is the function of temperature) because the oxidation rate is different. The equation for this is Equation (1):

H_2_ + ½O_2_ → H_2_O (1)

When any reduced gas reaches the tin dioxide surface, the oxidation reaction is performed first, the oxyanions adsorbed on the surface are removed, the positions left behind are replaced by the oxygen in ambient air, forming oxyanions again. This action is favorable for the oxidation of gas. When the reaction rate of the oxidation of gas reaches equilibrium with that of the replacement of oxyanions, the material surface reaches a stable state. This state is the equilibrium concentration of oxyanions adsorbed on the material surface. The micro hydrogen sensors published in prior documents require higher operating temperature for sensing [15,16]. Although SnSe film can be sensed at room temperature, the sensitivity is low [16], or uses an ultra-thin chemically sensitive layer as the sensing layer [17]. For sensing inside the proton battery, the operating temperature and volume of micro hydrogen sensor shall be reduced greatly, so as to reduce the influence on the performance of proton battery.

## 3. Process Development of Flexible Micro Hydrogen Sensor

**Figure 1:**
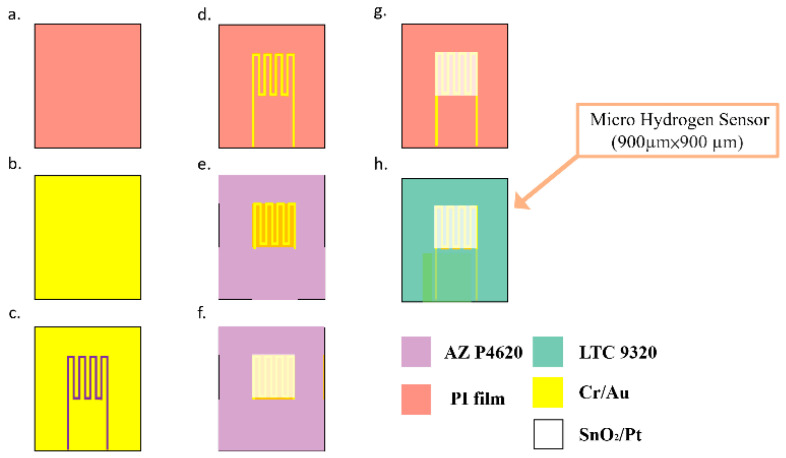
Process diagram of flexible micro hydrogen sensor.

**Figure 2:**
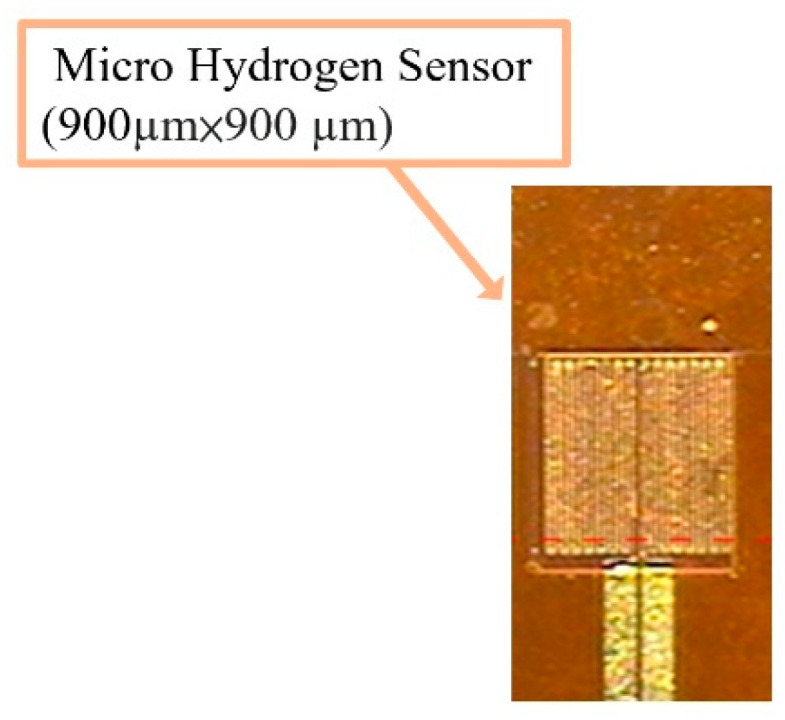
Optical micrograph of flexible micro hydrogen sensor.

This study successfully used MEMS technology to develop a micro hydrogen sensor on the flexible substrate of polyimide film (PI). The lift-off process is used to make the hydrogen sensing layer. Stannic oxide has to be used for the sensing layer with platinum as a catalyst layer. First, coat the positive photoresist (AZ® P4620, Microchemicals GmbH, Ulm, Germany) on the film and use a photomask (only hydrogen sensing area are exposure) exposure with mask aligner. Immerse in the developer to remove the photoresist on the hydrogen sensing area. Evaporate stannic oxide and platinum by using the electron beam evaporator. Wash it with acetone and methanol to remove the remaining photoresist, then wash with deionization water and dry it on a hot plate. The next step is vapor deposition, and the entire vapor deposition process is performed at a plating rate of 0.1 Å/s. First deposit 50, 100, 150 nm thick SnO_2_ as the gas sensor layer, and then deposit 250 Å thick Pt as the catalyst layer to complete the gas sensor. The fabrication process approximately comprises the following steps. The fabrication process is shown in Figure 1:

- The substrate of PI film is cleaned with organic solutions acetone and methanol, the residual methanol is removed by deionized (DI) water and the surface dust and residual oil and fat are removed, so as to enhance the adhesive ability of thin film metal.
- The Cr is evaporated by E-beam evaporator (Junsun, Taipei, Taiwan) as the adhesion layer of Au and PI film, the adhesion of Au and PI film is enhanced, and the deposition of 1000 Å thick Au is completed at a deposition rate of 0.1 Å/s.
- The AZ P4620 (positive photoresist) is spin coated; the electrode pattern of the micro hydrogen sensor is defined by exposure and development.
- The Au is etched by Au etching solution (Type-TFA, AppliChem Technology, Miaoli, Taiwan), and the Cr is etched by Cr etching solution (Cr-7T, AppliChem Technology, Miaoli, Taiwan); the photoresist as etching mask is removed by Remove 1165.
- The AZ P4620 is spin coated again as the mask for evaporation, and the pattern of micro hydrogen sensor is defined by exposure, and then the required pattern is developed by a developer.
- The SnO_2_ is evaporated by E-beam evaporator on the surface of the defined pattern as a gas sensing layer, and the Pt is evaporated as a catalyst layer.
- The sample is put in the photoresist remover (Remove 1165, MicroChemicals, Hsinchu, Taiwan) at 80 °C, kept still for about 20 min, then the photoresist mask is lifted off and cleaned with acetone and methanol (Minyung, Taoyuan, Taiwan).
- Finally, LTC 9320 (negative photoresist)( MicroChemicals, Hsinchu, Taiwan) is spin coated on the flexible micro hydrogen sensor to complete the protection layer. The optical microphotograph is shown in Figure 2.

## 4. Test and Correction of Flexible Micro Hydrogen Sensor

**Figure 3:**
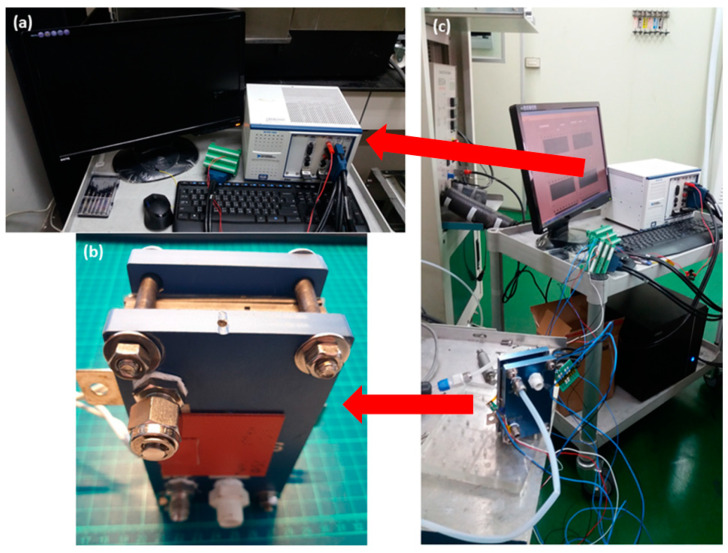
(**a**) National Instruments (NI). (**b**) Fuel cell testing machine. (**c**) The micro hydrogen sensor is tested and calibrated.

The purpose of performance testing of flexible micro hydrogen sensor is to know the sensing condition and efficiency of micro hydrogen sensor and to find out the parameters of operation and process optimization, so as to manufacture a low-temperature operated micro hydrogen sensor. The micro hydrogen sensor is tested and corrected using a fuel cell testing machine, as shown in Figure 3. The fuel cell testing machine can supply different gases, set up gas flow and heat gas. Therefore, the micro hydrogen sensor is embedded in the runner plate of proton battery, the runner of proton battery is used as a closed chamber for test. First of all, the micro hydrogen sensor is connected to the NI PXI 2575 data acquisition unit (Shining, Hsinchu, Taiwan) of a computer to detect the resistance change, the oxygen at constant temperature and constant flow is supplied, so that the surface of micro hydrogen sensor can adsorb O^−^, and then the hydrogen at constant temperature and constant flow is supplied; as the hydrogen carries the O^−^ away from the surface of the micro hydrogen sensor, the resistance of micro hydrogen sensor decreases. The hydrogen is detected according to the resistance difference between different gases.

**Figure 4:**
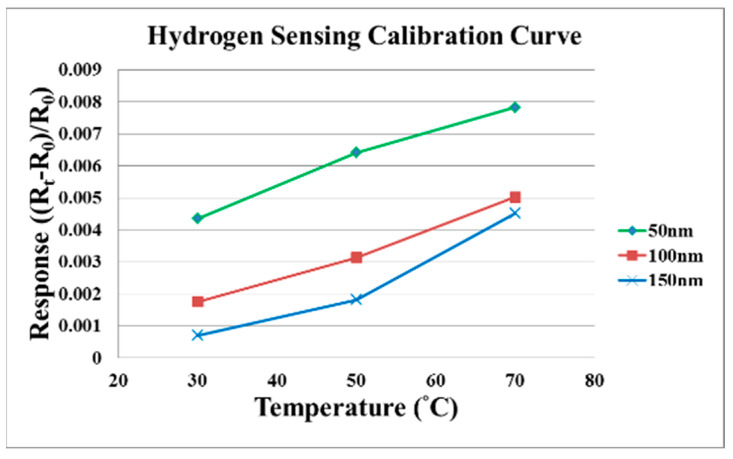
The film thickness of SnO_2_ evaporated at different temperatures (50, 100, 150 nm).

**Figure 5:**
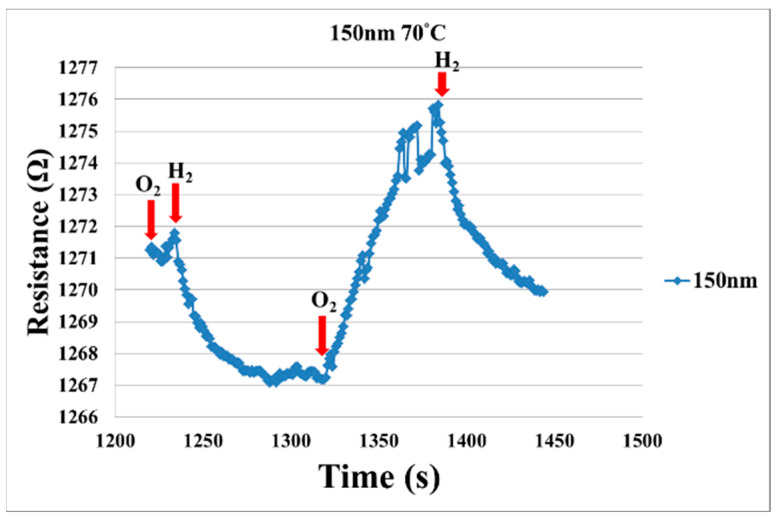
Resistance changes when oxygen and hydrogen are admitted into micro hydrogen sensor with SnO_2_ in film thickness of 150 nm at 70 °C.

Figure 4 shows the film thicknesses (50, 100, 150 nm) of SnO_2_ evaporated at the same temperature. The sensitivity to hydrogen is different, the SnO_2_ with film thickness of 50 nm has the best sensitivity, so the micro hydrogen sensor embedded in the proton battery is fabricated by evaporating SnO_2_ with a film thickness of 50 nm. Figure 5 shows the resistance changes when the oxygen and hydrogen are admitted into the micro hydrogen sensor evaporated with SnO_2_ in film thickness of 150 nm at 70 °C; the resistance changes when the O^−^ is adsorbed on the surface of SnO_2_ and the hydrogen is carried away.

## 5. Hydrogen Sensing Inside Proton Battery

**Figure 6:**
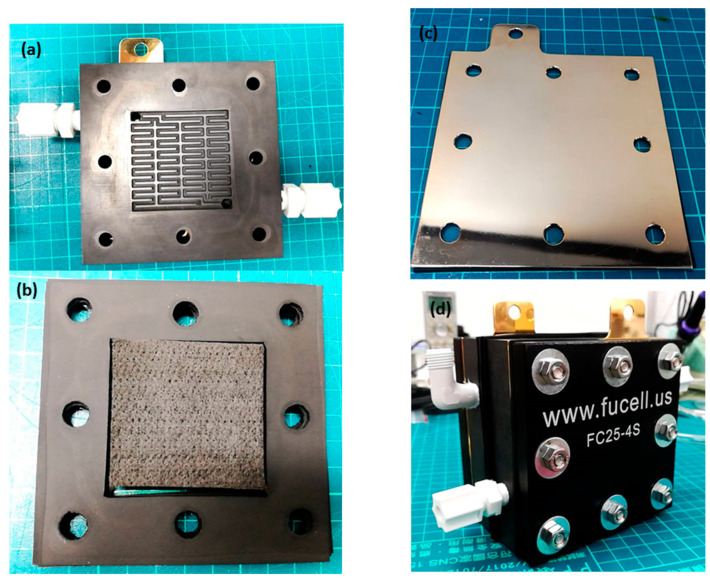
Proton battery. (**a**) Bipolar plate. (**b**) MEA. (**c**) Metal current collector plate. (**d**) Finished product.

**Figure 7:**
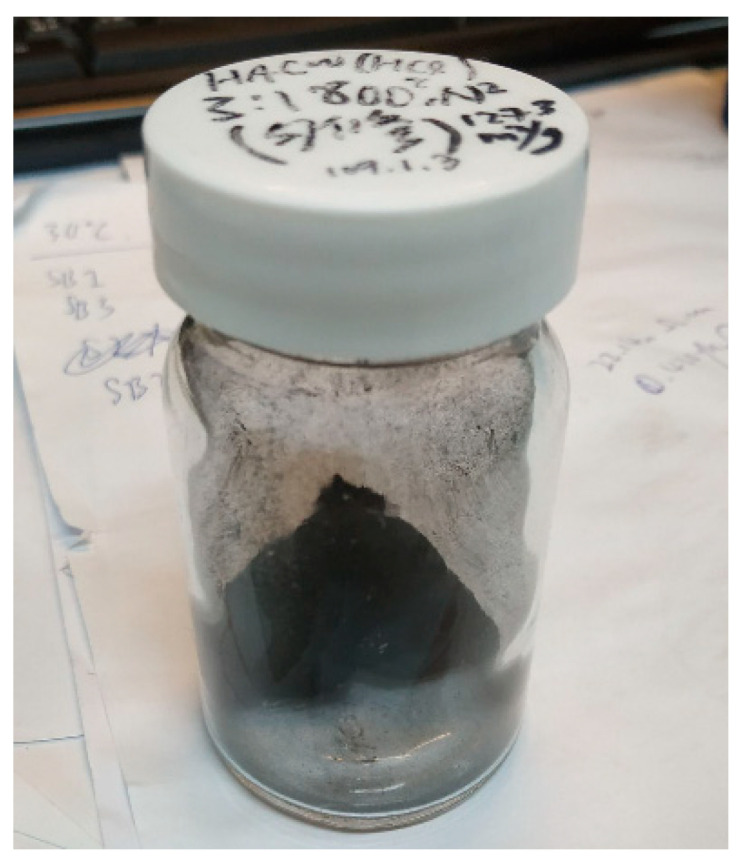
End product of activated carbon.

The technology of the proton battery is built on the fuel cell, so the materials are about the same; the entity is shown in Figure 6. The difference is the middle carbon layer, the specific surface area (Brunauer–Emmett–Teller area size) will influence the hydrogen storage capacity of the proton battery. The activated carbon from two companies has been studied; it was found that simple cleaning of activated carbon cannot improve the specific surface area; when the KOH at different concentrations is heated to 800 °C for activation, the specific surface area is enlarged a lot. The findings show that the activated carbon from different companies has different KOH concentration ratios for obtaining the maximum value of a specific surface area, and when the concentration ratio generating the maximum value is exceeded, the specific surface area is reduced on the contrary, as shown in Table 1. Finally, the KOH concentration ratio 3:1 of Company A is used for the subsequent experiment, the end product of activated carbon is shown in Figure 7.

**Figure 8:**
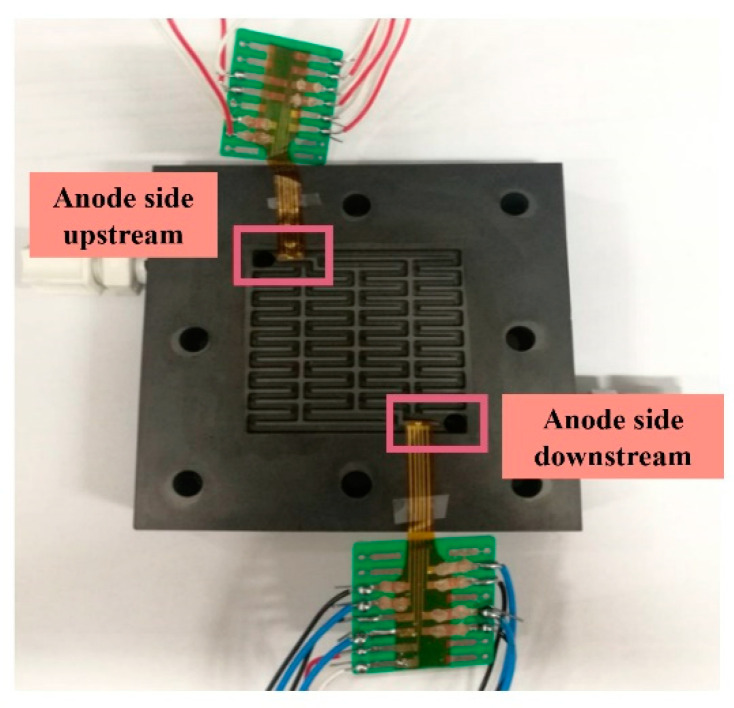
Stereogram of micro hydrogen sensor embedded in proton battery.

**Figure 9:**
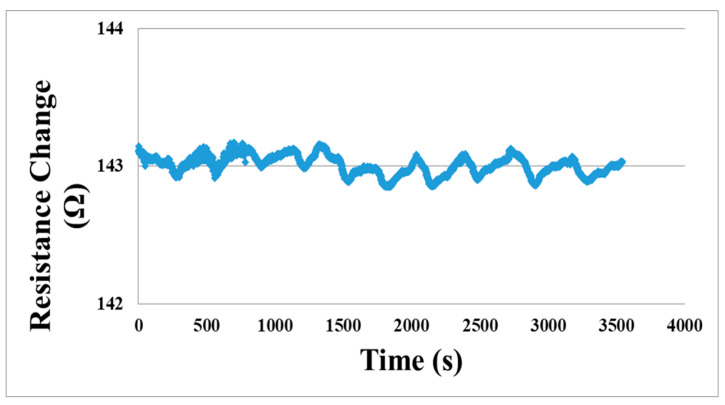
Real-time detection of hydrogen inside proton battery.

The micro hydrogen sensor is embedded in the proton battery, as shown in Figure 8. The activated carbon powder is put in the runner on the hydrogen side before assembly. After assembly, the sulfuric acid is added to the hydrogen side through the upstream inlet. The measuring methods include the charging mode and discharging mode. The micro hydrogen sensor is used for analyzing the internal data and proton battery, monitoring the variation of hydrogen instantly (distribution pattern in battery), and detecting the generation of hydrogen instantly. Figure 9 shows the resistance change instantly measured by the micro hydrogen sensor on the hydrogen side of the proton battery.

## 6. Conclusions

This study successfully developed a low-temperature flexible micro hydrogen sensor by using MEMS technology, which was embedded in a proton battery for real-time hydrogen sensing. This flexible micro hydrogen sensor is characterized by resistance to an electrochemical environment and low-temperature real-time measurement, and it can be placed in any position inside the proton battery.

## Figures and Tables

**Table 1:** Activated carbon specific surface area.

Laboratory Sample	Specific Surface Area
Original activated carbon of Company A	618.2029 m^2^/g
KOH concentration ratio 3:1 of Company A	1326.9233 m^2^/g
KOH concentration ratio 4.5:1 of Company A	1285.9408 m^2^/g
Original activated carbon of Company B	822.8134 m^2^/g
Company B after cleaning	846.6372 m^2^/g
KOH concentration ratio 4.5:1 of Company B	1025.9340 m^2^/g
KOH concentration ratio 7:1 of Company B	1173.1066 m^2^/g
